# Supplementary material for: Development and validation of a model for individualized prediction of hospitalization risk in 4,536 patients with COVID-19
Source: PLoS One. 2020 Aug 11;15(8):e0237419. doi: 10.1371/journal.pone.0237419 (PMC7418996; doi:10.1371/journal.pone.0237419)
Supplement: Attachment — Submitted filename: response to reviewers minor revision.docx [file pone.0237419.s003.docx]

July 16, 2020

Response to reviewer:

PONE-D-20-11909R1
Characteristics, outcomes, and individualized prediction of hospitalization risk in 4,536 patients with COVID-19.
PLOS ONE

Dear Dr.Orueta,

Thank you for the favorable review of our revised manuscript. We submit here the requested minor revisions with:

1- your request to add the tripod checklist and make the corresponding minor revisions in the manuscript, and
2- the only suggestion from the reviewer (reviewer #2) to add a new reference (now reference#28).

Looking forward to hopefully an expeditious final decision.

Best regards

Lara Jehi, MD, MHCDS and Mike Kattan, PhD
